# Measuring the physical activity in the EU using a fuzzy hybrid synthetic index and an ordered probit model

**DOI:** 10.3389/fspor.2025.1582658

**Published:** 2025-07-31

**Authors:** Juan Carlos Martín, Pedro Moreira

**Affiliations:** Institute of Tourism and Sustainable Economic Development, University of Las Palmas de Gran Canaria, Las Palmas de Gran Canaria, Spain

**Keywords:** physical activity, sport participation, walking, moderate exercise, eurobarometer, fuzzy-hybrid analysis

## Abstract

**Introduction:**

Physical activity can be measured by different attributes, such as sports activities, moderate exercise, or even walking time. The most recent Eurobarometer on Sport and Physical Activity included nine questions that permit physical activity measurement at the EU.

**Methods:**

The study uses a Fuzzy Hybrid Analysis approach to calculate a synthetic index that measures the physical activity of EU citizens. The method is applied to the dataset obtained from a survey administered to a total of 26,578 respondents who represent the EU. Nine items measure the physical activity latent variable with an answer format based on three different semantic ordinal point scales.

**Results:**

The method provides a synthetic indicator at aggregated and individual levels. Seventeen covariates were used to analyze the main determinants of physical activity, particularly gender, age, education, social class, and political orientation.

**Discussion:**

The results reveal that certain covariates influence the latent variable under study, providing interesting insights to inform the development of targeted programs that reduce physical inactivity in the EU.

## 1 Introduction

The importance of physical activity and sport in promoting health, well-being, and social cohesion is widely recognized for its role in controlling chronic non-contagious diseases such as hypertension, diabetes, coronary heart disease, obesity, and certain cancers. All these ailments increase the risk of mortality and mental health problems, affecting citizens' quality of life. Lack of or weak physical activity implies high economic costs, social disparities, and detriment to public health (1, 2).

Despite the promotion by international organizations, an estimated 27.5% of the global adult population fails to meet the recommended physical activity standards, significantly impacting public health issues. Several studies report significant differences in inactivity depending on gender, age, geographic area, and income level. Both women and older adults tend to have lower rates of physical activity, partly due to socio-cultural barriers and access to sports facilities (3).

In the present study, we analyze the physical activity of European Union (EU) citizens based on data obtained from the “Special Eurobarometer 525—Sport and Physical Activity”, a survey administered to more than 26,000 people in the 27 member states of the EU. Due to its large sample size and detailed socio-demographic clustering, this information provides in-depth insights into physical activity practices, including cultural and economic factors, as well as personal preferences (4).

The methodology is based on applying the multi-criteria technique TOPSIS (Technique for Order of Preference by Similarity to Ideal Solutions) with the perspective of Fuzzy Hybrid Analysis. The primary purpose of this approach is to address the ordinal and semantic nature of the responses more realistically and robustly (5). The method also handles the information vagueness more adequately than other traditional methods (6). In addition, the well-grounded properties of the fuzzy logic algebra provide an adequate framework for the construction of the aggregated synthetic indicators (7).

After obtaining this synthetic index, we applied an ordinal regression econometric model (ordered probit) to determine which covariates have a significant influence on the probability that an individual will be highly sedentary. Among the variables included in the selection are those related to age, level of education, citizens' perception of their EU membership, social class, gender, urban/rural area of residence, and purchasing power. The variety of these variables enables a multidimensional analysis that encompasses economic, socio-demographic, and personal attitudes and perceptions.

The main findings indicate a marked geographical polarization across the EU: northern and central nations (Finland, the Netherlands, Estonia, and Denmark) emerge as having higher physical activity; in contrast, southern and eastern regions (Portugal, Greece, Italy, and Poland) tend to have lower levels of physical activity. These differences may be associated with cultural elements (lower use of active transport and habits) and with government policies and urban infrastructure (existence of bicycle routes, pedestrian areas, and facilities for accessible sports spaces).

Furthermore, it is corroborated that older individuals and those with a lower educational and economic level have a significantly higher tendency towards sedentary lifestyles. However, young people, the upper-middle classes, and people with higher levels of life satisfaction tend to have higher rates of physical activity. Additionally, a sense of belonging to the community and a positive perspective on the EU's future are associated with a higher level of motivation to be physically more active.

We conclude that these findings underscore the need for policies promoting physical activity in Europe to be tailored to specific population groups and to account for each country's unique cultural and socio-demographic characteristics. The analysis methodology used, combining fuzzy logic with TOPSIS and a structured probit model, has proven to be particularly useful in revealing nuances that might be hidden in conventional measurements. In the context of EU and local government initiatives to reduce sedentary lifestyles, this analytical approach could be an efficient tool for managing decisions and developing more focused and effective public health strategies and sports policies.

## 2 Literature review

Physical inactivity has been associated with various pathologies, including obesity, overweight, and different health conditions associated with prolonged sedentary behavior (8). Indeed, it has been estimated that sedentary behavior contributes to between 6% and 10% of all deaths (9). Furthermore, it has been demonstrated that inactivity has a detrimental impact not only on the physical health of the population but also on its mental health and quality of life (10).

The promotion of physical activity and the reduction of sedentary behavior are considered essential for the prevention of non-contagious diseases, reduction of mortality and improvements in quality of life (11–13). This issue has gained significant attention from international organizations and governments (10–13).

Despite the systematic promotion of regular physical activity by various international organizations, it is estimated that 27.5% of the global adult population does not meet the recommended minimum levels of exercise (3). This reveals a significant health challenge: sedentary lifestyles not only increase the risk of noncommunicable diseases, but also have considerable economic costs for health systems and an impact on social inequality.

Initiatives such as the Global Strategy on Diet, Physical Activity and Health of the WHO—World Health Organization in 2004, the Global Action Plan for the Prevention and Control of Non-Contagious Diseases of 2013, or the Global Action Plan on Physical Activity (GAPPA) 2018-2030 highlight the need for integrated approaches to create favorable environments that facilitate the regular practice of physical activity.

Regarding the topic of study, we highlight research by Guthold et al. (3) on identifying patterns of inactivity not only at the global level but also stratified by gender, age, geographical regions, and national income. The authors collected and standardized data from 358 surveys conducted between 2001 and 2016, with an unprecedented sample of 1.9 million adults in 168 countries. In addition, by applying validated measurement instruments such as the International Physical Activity Questionnaire (IPAQ) and the Global Physical Activity Questionnaire (GPAQ), information on physical activity at work, at home, during transportation, and during leisure time could be gathered for the first time. The analysis was disaggregated by gender and income level, using multilevel mixed models that incorporated variables such as urbanization, educational level, and belonging to one of the nine macro-regions defined by the WHO. The author found inequalities and challenges public health programs face, providing governments with well-targeted policy design.

Overall, the study describes how by 2016, approximately 27.5% of the adult population worldwide did not achieve WHO-recommended levels of physical activity, corresponding to at least 150 minutes of moderate activity per week, 75 minutes of vigorous activity, or an equivalent combination (14). It was also found that income, urbanization dynamics, transport use, and sedentary jobs affected the levels of physical activity (15). High-income countries have a prevalence of inactivity more than twice as high as that observed in low-income countries, a finding that could be explained by an increase in transport motorization, a sedentary employment-oriented lifestyle, increasing urbanization, and a displacement of traditional and more physical activities (16, 17).

Regarding gender differences, in 159 out of 168 countries, women exhibited higher levels of inactivity than men, with gaps of at least 10 percentage points in 65 countries (in 9 of them even exceeding a 20-point difference). There were significant differences between men and women, with a gap of more than 8 percentage points globally, with women being the most inactive in most regions. This disparity could be due to socio-cultural factors that constrain women's participation in recreational or sports activities, as well as their greater dedication to care roles and household duties, which are not always classified as formal physical activity (14).

The success of interventions such as improving pedestrian infrastructure, promoting cycling or public transport, developing urban parks and green spaces, as well as consciousness-raising campaigns, depends to a large degree on political willingness and the cross-cutting cooperation of government agencies, the private sector, and citizens (16). From a sociological perspective, it is essential to understand that the adoption of active habits is directly related to socio-economic, cultural, and symbolic factors; therefore, individual recommendations alone are not enough. There is also a clear relationship between income inequality and health (18).

There are many programs scattered worldwide, aligned with the Global Action Plan on Physical Activity 2018-2030 promoted by WHO (15), aiming to reduce physical inactivity levels by 15% by 2030. To achieve this goal, a multi-sectoral strategy involving governments, health systems, local organizations, enterprises, and community groups appears essential. The success of such physical activity promotion policies depends to a large degree on how the supply of sports infrastructure and health education is aligned with the conditions of inequality. This is the only way to overcome sedentary tendencies resulting from modern life by promoting physical activities as an accessible, culturally meaningful, and valued practice for large population sectors.

Klepac Pogrmilovic et al. (19) proposed a framework of accurate definitions to categorize and evaluate physical activity policies, which they named “Comprehensive Analysis of Policy on Physical Activity” (CAPPA). They identified a series of variables that affect policies related to physical activity, for example, health, sport, transport, environment, job/employment, education, tourism, urban planning, public finances, and research, among others (15). Thus, they generated a comprehensive approach to studying physical activity policies that promote healthy behaviors. This conceptual framework could guide and focus resources, identifying gaps, opportunities, and challenges of the existing physical activity promotion policies. The modular structure of the framework would enable its adaptability to local contexts, allowing for the comparability of results across different countries.

We conclude the literature review by focusing on the analysis of two key determinants: age and education. It is essential for older adults to engage in activities to maintain their physical health, mental well-being, and social well-being. Older adults often face barriers to accessing suitable physical activity programs, including mobility difficulties, chronic diseases, and a lack of personalized activities (20–22). For children, regular and consistent physical exercise is also essential for optimal physical and mental development. They are a particularly vulnerable group due to the absence of appropriate infrastructure, the lack of safe and affordable exercise programs, and sedentary lifestyles promoted by the increasing use of digital technologies. Children's vulnerability in this area can have long-term impacts on their health, including obesity, metabolic disturbances, and difficulties in developing motor and social skills (23, 24).

Low childhood physical activity is a growing concern, given its direct association with the prevalence of obesity, cardiovascular problems, and diabetes (2, 25). The WHO recommends that children aged 5–17 years engage in at least 60 minutes of moderate to vigorous physical activity per day. However, multiple studies indicate that most children do not reach this level (22, 26). This problem is not only explained by individual factors such as motivation or skills, but also by characteristics of the socio-cultural environment and, in particular, school environments (27, 28). The existence of school policies promoting physical activity, along with available facilities and opportunities for physical activity (e.g., through recreational breaks, extracurricular sports, and sports competitions), can substantially support schoolchildren's participation in regular physical activity (29).

Finally, education emerged as a strong, positively correlated factor. Increased knowledge of the benefits of physical activities, combined with better socio-economic conditions, facilitates the overcoming of obstacles (Janke et al., 2006). Living in one's own home and being employed were also positively associated with activity, indicating the complexity of older people's life trajectories and their socio-economic circumstances. While there was no significant main effect of gender on activity, the interaction with “fear of injury” resulted in women being particularly vulnerable to sedentary lifestyles when this perception was present (30).

## 3 Data

The special Eurobarometer 525 was commissioned by the Directorate-General for Education, Youth, Sport and Culture at the European Commission to explore public opinion about sport and physical activity, covering the following topics: (1) Frequency and levels of engagement in sport and other physical activity; (2) Places where citizens engage in sport and other physical activity; (3) Europeans' motivators and barriers to sports participation; (4) Opportunities for sports participation in citizens' local areas; (5) Europeans' engagement in volunteering in sport; (6) Impact of COVID-19 on the frequency of sport and physical activity; (7) Awareness of the impact of sport and physical activity on the environment and perceptions of measures taken to support the environment; and (8) Opinions about gender equality issues in sport and physical activity.

The current study primarily addresses questions related to the first topic, analyzing a synthetic index that measures the physical activity of EU citizens. The survey was conducted among 26,578 EU citizens by the Kantar network across the 27 Member States of the European Union between 19 April and 16 May 2022. The sample was representative of the EU, and participants were drawn from various social and demographic categories. For more details and technical specifications of the survey, please refer to the European Commission (4).

The physical activity scale is based on nine indicators that correspond to the following questions of the survey: (QB1): How often do you exercise or play sport? By “exercise”, we mean any form of physical activity which you do in a sports context or sport-related setting, such as swimming, training in a fitness center or a sports club, or running in the park; (QB2): And how often do you engage in other physical activities such as cycling from one place to another, dancing, gardening, etc.? By “other physical activity”, we mean physical activity for recreational or non-sport-related reasons; (QB3): In the last 7 days, on how many days did you do vigorous physical activity like lifting heavy things, digging, aerobics or fast cycling?; (QB4) In general, on days when you do a vigorous physical activity, how much time do you spend at it?; (QB5) In the last 7 days, on how many days did you do moderate physical activity like carrying light loads, cycling at normal pace or doubles tennis? Please do not include walking; (QB6): In general, on days when you do a moderate physical activity, how much time do you spend at it?; (QB7) In the last 7 days, on how many days did you walk for at least 10 minutes at a time?; (QB8): In general, on days when you walk for at least 10 min at a time, how much time do you spend walking?; (QB9): How much time do you spend sitting on a usual day? This may include time spent at a desk, visiting friends, studying, or watching television.

One of the issues to be discussed in the methodology section is that the answer format of the questions is different in format and the number of points chosen for the response. For example, the answer format of the questions QB1 and QB2 was based on a 6-point ordinal time per month scale as follows: (1) Never; (2) Less often than 1–3 times a month; (3) 1–3 times a month; (4) 1–2 times a week; (5) 3–4 times a week; and (6) 5 times a week or more. Meanwhile, for the questions QB3, QB5, and QB7, the answer format was based on an 8-point ordinal time per week scale as follows: (1) Never; (2) 1 day; (3) 2 days; (4) 3 days; (5) 4 days; (6) 5 days; (7) 6 days; and (8) 7 days. For the questions QB4, QB6, and QB8, the answer format was based on a 6-point ordinal time per day as follows: (1) Never; (2) 30 min or less; (3) 31 to 60 min; (4) 61 to 90 min; (5) 91 to 120 min; and (6) More than 120 min. Finally, for QB9, the answer format scale is based on a 10-point ordinal time per day as follows: (1) More than 8 h and 30 min; (2) 7 h 31 min to 8 h 30 min; (3) 6 h 31 min to 7 h 30 min; (4) 5 h 31 min to 6 h 30 min; (5) 4 h 31 min to 5 h 30 min; (6) 3 h 31 min to 4 h 30 min; (7) 2 h 31 min to 3 h 30 min; (8) 1 h 31 min to 2 h 30 min; (9) 1 h 1 min to 1 h 30 min; and (10) 1 h or less.

In summary, we have four different answer format scales based on ordinal times at different periods with six, eight and ten points. The direction of the scale for all the items was consistent, meaning that higher figures are aligned in all cases with more physical activity, either by participating in sports, vigorous or moderate physical activities, walking, or staying seated less.

The physical activity synthetic indicator (PASI) will be constructed at the individual level and aggregated level using fifteen different covariates: country, age, life satisfaction, personal opinion about whether belonging to the EU is good or bad for the country, opinion about the future of the EU, opinion about if the national countries should help refugees, political orientation, marital status, education, gender, household location, having or not difficulties in paying bills, social class, opinion about the future for the next EU generations, and community type. Thus, it would be possible to analyze to what extent PASI is affected by these factors. Thus, we significantly extend the analysis of each variable studied in the Eurobarometer in terms of the covariates used. For example, in the final report (4), the answers given to QB2 were only cross-tabulated with country, gender, age, education, and difficulties in paying bills.

## 4 Methodology

PASI will be obtained at the individual level using the Technique for Order of Preference by Similarity to Ideal Solutions (TOPSIS) proposed by Hwang and Yoon (31). The method chooses the individuals who have carried out the most and the least physical activity in the sample and compares each individual with these two by calculating the relative distance to both ideal solutions. Unfamiliar readers in TOPSIS methodology often find it challenging to assimilate the negative ideal solution (or anti-ideal solution) as an “ideal” reference point, especially when understood in our context of minimizing the physical activity. It is true that linking ideal with low physical activity seems to be counterintuitive. However, this negative ideal solution and the positive ideal solution (those individuals with the highest physical activity) are only chosen as reference points to rank all the individuals' physical activity.

Thus, the synthetic indicator is obtained by calculating the ratio between the distance to the least physical activity over the sum of distances to both ideal solutions. The idea is that when the ratio is closer to one, the individual is closer to the individual who has carried out the most physical activity.

TOPSIS is still one of the most used multi-criteria decision-making methods and was extended to fuzzy sets using a fuzzy-hybrid analysis (FHA) in which the attribute values are represented by fuzzy numbers instead of crisp numbers (32). Thus, a more robust analysis can better address the uncertainty of Likert, semantic, or ordinal scale (33). This hybrid approach has been applied in different fields, such as selecting the best shopping websites (34), fish consumption in the EU (5), commuter satisfaction in Central Europe (35), analyzing service quality in the MICE industry (7), tourist destination competitiveness (36), transshipment site selection (37), and wind power potential plants (38). More recently, the application has also gained popularity in social science to measure attitudes toward migrants (39), or to analyze citizens' national identity (6).

We omit the mathematical formulation for the ease of exposition and because there are excellent manuals that cover the methods used in the study (40–42). Nevertheless, for the sake of replicability, a crucial aspect of this study, the conversion of ordinal scales to Triangular Fuzzy Numbers (TFNs) was conducted according to the following procedure: 6-point scales (1 = (0 0 30); 2 = (5 20 35); 3 = (25 40 55); 4 = (45 60 75); 5 = (65 80 95); v6 = (70 100 100)); 8-point scales (1 = (0 0 25); 2 = (5 15 25); 3 = (20 30 40); 4 = (35 45 55); 5 = (45 55 65); 6 = (60 70 80); 7 = (75 85 95); 8 = (75 100 100)), and 10-point scale (1 = (0 0 10); 2 = (0 10 20); 3 = (10 20 30); 4 = (20 30 40); 5 = (27.5 40 52.5); 6 = (47.5 60 72.5); 7 = (60 70 80); 8 = (70 80 90); 9 = (80 90 100); 10 = (90 100 100)). It can be seen that in all the cases, the intersection of the converted TFNs of any pair of consecutive points is not empty. This is the essence of the nature of the fuzzy logic when the human knowledge is limited and precise information on significant statements about our behavior does not exist (43).

In our study, the conversion process was guided by established practices and interpretations within the fuzzy set theory literature relevant to social science data (44). The main idea is to represent the inherent imprecision and vagueness associated with ordinal scales in a way that is both meaningful and preserves the underlying order of the categories. Furthermore, other empirical studies have specifically addressed the potential impact of different conversion approaches on the robustness of results within the context of fuzzy-hybrid TOPSIS. For example, Martín et al. (45) showed that, while the specific TFN values assigned can influence the absolute scores, the relative ranking and the overall conclusions derived from the fuzzy-hybrid TOPSIS approach tend to be quite robust across a range of sensible conversion strategies.

One of the steps of TOPSIS consists of normalizing the decision matrix. This is done to ensure that all the criteria are comparable. In the current study, it would be challenging to compare ordinal scales from different time periods, such as hours in days, days in weeks, or times per month. There are a number of ways to normalize a decision matrix (46), but in the current study, this is resolved by the conversion of the ordinal scales into TFNs that belong to the universe of discourse in the range [0, 100]. The linearity between the points in an ordinal scale is also a relevant issue, that can be partly resolved by using TFNs. It is unreasonable to think that there is the same distance between never, less often than 1–3 times a month, and 1–3 times a month. Less often than 1–3 times a month can be either 1 time every two months or 2 times per term. In the case of the highest scores, it can be seen that, for example, in the first scale, 5 times per week or more would be comparable to at least 20 times per month, in comparison with the ordinal values considered as 2 and 6. This issue is known as the non-linear scaling effect (47).

Once PASI was calculated, we preferred to discretize it, as the aim of the study is not to explain physical activity but to characterize the factors that affect two main categories of individuals: those who are quite sedentary in comparison with the most active segment. Thus, the PASI distribution is categorized according to the five quintiles to estimate an ordered probit model, where *y* denotes the random variable whose value ranges from 1 to 5, corresponding to each quintile according to PASI values. Thus, the model uses an auxiliary latent variable y* determined by:

(1) $$y^{*}=x\beta +\varepsilon$$

Where x is a vector formed, in principle, by the fifteen variables explained in the data section that are included in the model as the determinant factors that affect PASI, beta is the vector of parameters to be estimated by the model, and epsilon is the error term that distributes as a standard normal distribution. The model also determines four threshold parameters $\eta_{1}<\eta_{2}<\eta_{3}<\eta_{4}$, which permit to linking the observed dependent variable with the unobserved latent variable as follows:

(2) $$\begin{array}{rl} & y\;=\;1\;\mathrm{if}\;y^{*}\leq \eta_{1}\\ & y\;=\;2\;\mathrm{if}\;\eta_{1}<y^{*}\leq \eta_{2}\\ & y\;=\;3\;\mathrm{if}\;\eta_{2}<y^{*}\leq \eta_{3}\\ & y\;=\;4\;\mathrm{if}\;\eta_{3}<y^{*}\leq \eta_{4}\\ & y\;=\;5\;\mathrm{if}\;\eta_{4}<y^{*} \end{array}$$

All the parameters are estimated by maximizing, as usual, the log-likelihood function, which is consistent and asymptotically normal. The probability of observing a particular outcome for (1≤j≤5) is given by

(3) $$\begin{array}{rl} P(y_{i}=j|x_{i})= & \;P(\eta_{\;j-1}\leq \;y_{i}^{*}\leq \eta_{j})\\ = & \;P(\;\eta_{\;j-1}-x_{i}\beta \leq \varepsilon_{i}\leq \eta_{j}-x_{i}\beta )\\ = & \;F(\;\eta_{j}-x_{i}\beta )-F(\eta_{\;j-1}-x_{i}\beta ) \end{array}$$

Where F is the cumulative normal distribution function assumed for the error term, $\eta_{0}=-\infty ,\;\mathrm{and}\;\eta_{5}=\infty$. Then, we can write the log-likelihood function as follows:

(4) $$\log\;L\;=\;\sum_{i=1}^{N}\sum_{\;j=1}^{5}y_{ij}\log\;[F(\;\eta_{j}-x_{i}\beta )-F(\eta_{\;j-1}-x_{i}\beta )]$$

The log-likelihood is maximized with respect to the parameters of the distribution function and the cut thresholds. There are various specifications of categorical variables (normalization) in choice or ordinal models. The most used is that of the dummies normalization, but other normalizations like the effects code and Daly normalization, less common in the literature, are more informative regarding the comparisons that can be made. Interested readers are referred to the following excellent references on the topic (48–50). In the current study, we decided to normalize all the categorical variables with the normalization proposed by Daly et al. (48) to address interpretation issues with respect to the average EU citizen.

## 5 Results

Table 1 shows the ideal solutions, the representative of each value, and the percentage variation between the positive and negative ideal solutions. The figures and the representative values provide valuable information regarding which covariates can play a determinant role. It can be seen that most of the representative values correspond to the country covariate, so it can be easily inferred that PASI is highly affected by some cultural issues. Sterk & Bürgi (51) note that cultural environment can significantly influence physical activity patterns, confirming that country variable plays a significant role in interpreting the PASI index. The only two exceptions are presented in the negative ideal solution for the frequency of the sports activity and the time spent sitting, for which the representative values are the low-education segment and those who have refused to answer the social class self-classification question, respectively.

**Table 1:** Ideal solutions.

Indicator	A^+^	Representative	A^−^	Representative	Perc. Variation
Sport activity—frequency	57.04	FI—Finland	14.96	Education 15-	281.3%
Physical activity—frequency	74.98	NL—The Netherlands	14.89	PT—Portugal	403.5%
Vigorous physical activity—days last week	32.98	FI—Finland	9.66	PT—Portugal	241.4%
Vigorous physical activity—how much time	43.46	LV—Latvia	9.88	PT—Portugal	339.9%
Moderate physical activity—days last week	49.02	NL—The Netherlands	9.64	PT—Portugal	408.6%
Moderate physical activity—how much time	40.48	EE—Estonia	9.97	PT—Portugal	305.9%
Walked at least 10 minutes—days last week	74.54	ES -Spain	43.64	PL—Poland	70.8%
Walked at least 10 minutes—how much time	39.22	LV—Latvia	18.18	CY—Cyprus (Republic)	115.7%
Time spent sitting—usual day	65.60	MT—Malta	43.73	Social class Refusal (SPONT.)	50.0%

The last column of the table –the percentage variation between the ideal solutions figures- also provides interesting insights regarding in which indicators there is more or less heterogeneity in the sample. Thus, it can be seen that in the EU, there is more homogeneity in the time citizens spend sitting on a usual day, including time spent at a desk, visiting friends, studying, or watching television, and the number of days walking at least ten minutes in the last week. Interestingly, the representative values for the ideal solutions were seen in Spain (74.54) and Poland (43.64). On the other hand, more heterogeneity is observed in (QB2):—other physical activities such as cycling from one place to another, dancing, gardening, etc., i.e., physical activity for recreational or non-sport-related reasons, (QB4)—time spent doing a vigorous physical activity, and (QB5)—the number of days in the last 7 days, doing moderate physical activity like carrying light loads, cycling at a normal pace or doubles tennis, without including walking. Again, interestingly, the representative values for the positive ideal solution are observed in Northern countries –Latvia and the Netherlands, and Portugal was the representative value for the negative ideal solution for the three indicators. In the three cases, the percentage variation is around four hundred percent. This is concordant with the findings of Van Bottenburg (52) who found significant disparities in sport participation between different regions of the EU, with northern countries leading the physical activity levels.

Table 2 presents PASI for the total sample (EU27), and some covariates like country, age, life satisfaction, opinion on whether being a member state of the EU or not is a good thing, and the opinion on the future of the EU. The result of the EU27 was surprisingly 0.5, a value that can be used as a reference to analyze whether other segments have more or less physical activity than the average citizen in the EU. The country results determine a clear differentiation between the South (Portugal, Greece, Malta, and Italy), which, jointly with Poland, exhibits the least physical activity, and the North (Estonia, Sweden, Denmark, Finland, and the Netherlands), which exhibits the highest physical activity.

**Table 2:** PASI for some covariates.

Covariate	Segment	PASI	Covariate	Segment	PASI
-	EU27	0.500	Age	Age65+	0.410
Country	PT—Portugal	0.073		AgeRefusal	0.429
	PL—Poland	0.226		Age55–64	0.462
	GR—Greece	0.239		Age45–54	0.492
	IT—Italy	0.257		Age35–44	0.518
	MT—Malta	0.262		Age25–34	0.593
	CY—Cyprus (Republic)	0.271		Age15–24	0.710
	RO—Romania	0.282	Life Satisfaction	LS Not at all satisfied	0.257
	BG—Bulgaria	0.346		LS Not very satisfied	0.329
	HU—Hungary	0.389		LS DK (SPONT.)	0.402
	ES -Spain	0.451		LS Fairly satisfied	0.486
	IE—Ireland	0.461		LS Very satisfied	0.667
	HR—Croatia	0.469	Being a member state in the EU	EU MB DK (SPONT.)	0.364
	FR—France	0.473		EU MB Neither a good thing nor a bad thing	0.421
	AT—Austria	0.511		EU MB A bad thing	0.422
	BE—Belgium	0.523		EU MB A good thing	0.545
	DE-E Germany East	0.529		EU Future DK (SPONT.)	0.334
	CZ—Czech Republic	0.566	EU future	EU Future Very pessimistic	0.406
	SK—Slovakia	0.570		EU Future Fairly pessimistic	0.439
	LT—Lithuania	0.578		EU Future Fairly optimistic	0.544
	SI—Slovenia	0.628		EU Future Very optimistic	0.555
	DE-W—Germany—West	0.630			
	LU—Luxembourg	0.656			
	LV—Latvia	0.661			
	EE—Estonia	0.674			
	SE—Sweden	0.753			
	DK—Denmark	0.754			
	FI—Finland	0.797			
	NL—The Netherlands	0.805			

The age results also show a clear division between seniors (less physical activity) and young segments (more physical activity). Regarding life satisfaction, it can be seen that the two extremes are presented as not at all satisfied (least physical activity) and very satisfied (highest physical activity). Finally, a similar pattern is found for those who are pessimists or optimists on the future of the EU, being more physically active, the optimist group. In this regard, it is noteworthy that Mangra et al. (53) found a significant correlation between physical activity, general life satisfaction, and personal expectations about the future of the European Union. They also found that North-South and generational disparities directly influence the likelihood of adopting a more or less dynamic lifestyle.

Supplementary Table A1 reports the estimation results of the ordered probit model. It can be seen that most of the covariates are significant except for left-right political orientation and the community type, i.e., whether the citizen's household is in a city urban neighborhood, a suburb, or a rural community. These two results mean that physical activity does not depend on political orientation and the type of community in which citizens reside. This coincides with the results of Lira et al. (54) in their study on the irrelevant relationship between political orientation and possible solutions to physical inactivity. Out of these two covariates, the remaining thirteen covariates affect physical activity. The table also shows how the threshold coefficients were also significant.

Supplementary Table A2 shows the marginal effects of being in the first category of the endogenous variable, i.e., those who performed the least physical activity. The remaining marginal effects were computed and can be provided upon request. The estimated parameters offer significant insights into how variations in independent variables affect the outcomes related to physical activity levels. By analyzing these parameters, we can gain a deeper understanding of the factors that contribute to individuals becoming more or less physically active, shedding light on the complex relationships between various influences and their impact on overall activity levels.

Nevertheless, as our main objective was to characterize the EU citizen's profile as being sedentary, we have preferred to present only the results of the marginal effects of outcome 1. In addition, as the model contains more than one hundred coefficients, for ease of exposition, we present a table with the main drivers and barriers to being a sedentary person in the EU (Table 3).

**Table 3:** Drivers and barriers to be a sedentary person in the EU.

Barriers			Drivers		
Variable	Marg.Eff	Disc	Variable	Marg.Eff	Disc
FI—Finland	−14.4%	***	PT—Portugal	19.2%	***
NL—The Netherlands	−10.9%	***	Non binary	17.5%	**
LV—Latvia	−10.6%	***	PL—Poland	15.5%	***
EE—Estonia	−10.4%	***	MT—Malta	14.8%	***
Age15–24	−8.8%	***	CY—Cyprus (Republic)	11.4%	***
DK—Denmark	−8.2%	***	IT—Italy	10.2%	***
SE—Sweden	−7.9%	***	Education No full-time education	10.1%	***
LT—Lithuania	−6.9%	***	GR—Greece	9.4%	***
SK—Slovakia	−6.8%	***	RO—Romania	8.2%	***
DE-W—Germany—West	−5.7%	***	Social class DK (SPONT.)	5.5%	**
SI—Slovenia	−5.4%	***	Education Don't know	5.5%	***
Social class The higher class of society	−4.6%	**	Education 15-	5.4%	***
Education Still Studying	−3.9%	***	Education Refusal	5.2%	*
DE-E Germany East	−3.9%	**	Marital Status Widow	4.7%	***
CZ—Czech Republic	−3.7%	***	HU—Hungary	4.0%	***
LU—Luxembourg	−3.4%	**	IE—Ireland	3.9%	***
LS Very satisfied	−3.1%	**	BG—Bulgaria	3.8%	***
Age25–34	−3.1%	**	EU Future DK (SPONT.)	3.7%	***
Social class The upper middle class of society	−3.1%	***	LS Not very satisfied	3.5%	***
Education 20+	−2.7%	***	Age65+	3.5%	***
Rural area or village	−1.9%	***	LS Not at all satisfied	3.1%	**
Man	−1.6%	***	BE—Belgium	2.5%	**
Help Refugees Totally agree	−1.5%	***	Social class The working class of society	2.3%	***
			Diff. Paying bills Most of the time	2.2%	***
			HR—Croatia	1.7%	*
			Large town	1.7%	***
			EU Future. Life NextGen About the same	1.4%	***
			Help Refugees Tend to agree	1.4%	***
			Woman	1.4%	***
			Social class The lower middle class of society	1.4%	***
			Age55–64	1.3%	***
			Education 16–19	1.1%	***

Discussion: *** (*p* < 0.001); ** (*p* < 0.01); * (*p* < 0.05);. (*p* < 0.1).

It can be seen that the main barriers to being an EU sedentary citizen are being a citizen from the following countries: Finland, the Netherlands, Latvia, Estonia, Denmark, Sweden, Lithuania, Slovakia, Germany, Slovenia, the Czech Republic, and Luxembourg. As a general summary by country, it can be said that residing in the Northern EU makes one less likely to be a sedentary person. Other barriers are related to age, as citizens in the age groups 15–24 and 25–34 are less sedentary. Similarly, the upper classes of society –the higher and upper-middle- are also less sedentary. Education –still studying or highly educated citizens are also less sedentary than the average citizen. Citizens who are very satisfied with their lives are less sedentary, similar to citizens living in rural areas or villages, men and those who totally agree that governments must help refugees. López-Valenciano et al. (55) also found variations in sedentary lifestyles in the EU adult population between 2002 and 2017, with marked differences by age group and region. And in line with this analysis by highlighting the importance of country of residence, educational level and age as determinant and explanatory variables of physical inactivity.

On the other hand, the main drivers to be a sedentary person in the EU are characterized by being: from Portugal, Poland, Malta, Cyprus (Republic), Italy, Greece, Romania, Hungary, Ireland, Bulgaria, Belgium, or Croatia; non binary; low-educated (no full-time education, education 15, or education 16–19); no self-identified in any social class, or identified as the working class of society or the lower middle class of society; widows; respondents no knowing the EU future; not very satisfied or not at all satisfied with their lives; more than 65 years or between 55 and 64; respondents having difficulties in paying the bills most of the time; citizens living in large towns; respondents who think that in the future in the EU, the life for the next generation will be about the same; respondents tending to agree in that the government must help refugees; and women.

## 6 Conclusions

Growing evidence indicates that regular physical exercise helps prevent various chronic diseases and has a positive impact on the socio-economic context. Conversely, a lack of physical activity is associated with high health costs, increasing social inequality, and a declining quality of life. In this context, physical activity serves as a crucial component of public policies, necessitating multi-sectoral strategies that integrate health, urban planning, and education.

Several structural factors influence the adoption of either active or sedentary lifestyles. This conclusion is drawn from a study based on a physical activity survey conducted among citizens in the 27 countries of the European Union. We employed a methodology that combines fuzzy logic with the multi-criteria technique known as TOPSIS to develop the Physical Activity Synthetic Indicator (PASI) index. Additionally, we estimated an ordered probit model to assess the significance of socio-demographic, educational, economic, and attitudinal variables on having a more or less sedentary life.

Confirming territorial inequalities in Europe, mainly between north-south and, to a lesser degree, east-west. A higher rate of exercise and physical activity in northern countries could be attributed to the development of infrastructure for non-motorized mobility. Southern and some Eastern European countries are more sedentary, primarily due to cultural and climatic factors, as well as a possible lack of integrated physical activity promotion strategies. Geographical gaps reflect differences in the availability of physical activity environments and reflect citizens’ public policy priorities and physical activity traditions.

To analyze patterns of physical inactivity, it is relevant to consider socio-demographic variables such as age, social class, and educational level. Elderly adults are more likely to be in the lower PASI quintiles due to their functional limitations and lack of adapted exercise programs. People with lower levels of education are more likely to be sedentary due to less access to health information and more difficult employment conditions. Young people with higher education and from higher socio-economic groups tend to participate more in regular physical activities.

We also highlight the dimension of gender differences. Social care roles, domestic obligations, and difficulties reconciling work and family life may hinder regular participation in physical activities, leading to lower reporting rates. The sense of insecurity in public spaces and the limited availability of physical activities for women may also contribute to the gap, as well as cultural barriers.

The study found that life satisfaction and positive perceptions of the EU correlate positively with physical activities in the attitudinal and subjective dimensions. Physical exercise promotion campaigns, linked to optimism and collective well-being messages, can improve physical activity rates. Willingness to show solidarity, such as favoring countries helping refugees, is associated with lower levels of sedentary behavior, reflecting greater social openness and civic engagement in the greater common good.

From a public policy management perspective, we consider it necessary to design specific interventions targeted toward groups at higher risk of inactivity: the elderly, women, people with low levels of education, children, and residents in countries or regions with poor sports and non-motorized transport infrastructures. It is also important to encourage “walkability” in cities, promote efficient public transport, and create green spaces and safe areas for outdoor activities. Health promotion strategies should integrate interdisciplinary approaches, including urban planning, education, and social protection systems. Furthermore, policies and initiatives to reduce socioeconomic inequality could positively impact participation in physical activities and sports.

Finally, it is clear that physical inactivity is conditioned by several variables in each region of the EU and is not only a matter of individual choice or personal motivation. This contribution could provide an impulse for more targeted and equitable policies to promote a sustained increase in physical activity in Europe, addressing the implications of sedentary lifestyles for public health and social well-being.

Moreover, this study confirms the validity and usefulness of the hybrid fuzzy methodology combined with TOPSIS and the ordered probit model. TOPSIS facilitates the aggregation of multiple items into a synthetic indicator. The fuzzy logic mitigates the problems arising from the ordinal and subjective nature of the questionnaire questions. Identifying which covariates show statistically significant associations with the probability of belonging to a lower or higher activity stratum enables better evidence-based decision-making by providing a comprehensive understanding of the determinants of physical activity.

## Data Availability

The original contributions presented in the study are included in the article/Supplementary Material, further inquiries can be directed to the corresponding author.
