# Supplementary material for: Measuring the physical activity in the EU using a fuzzy hybrid synthetic index and an ordered probit model
Source: Front Sports Act Living. 2025 Jul 31;7:1582658. doi: 10.3389/fspor.2025.1582658 (PMC12352325; doi:10.3389/fspor.2025.1582658)
Supplement: Supplementary file 1 [file Table1.docx]

Supplementary Material

Table A1. Results of the ordered probit model

| **Variable** | **Estimate** | **Std..error** | **T.value** | **Pr...t..** | **Disc** |
| --- | --- | --- | --- | --- | --- |
| FR - France | -0.042628312 | 0.033165023 | -1.285339406 | 0.198673705 |  |
| BE - Belgium | -0.097298038 | 0.032079835 | -3.032996848 | 0.002421381 | ** |
| NL - The Netherlands | 0.431145149 | 0.034718819 | 12.4181975 | 2.082E-35 | *** |
| DE-W - Germany - West | 0.225601403 | 0.033401328 | 6.754264558 | 1.43562E-11 | *** |
| IT - Italy | -0.403313112 | 0.034295697 | -11.75987525 | 6.28271E-32 | *** |
| LU - Luxembourg | 0.133770837 | 0.047893368 | 2.793097324 | 0.005220598 | ** |
| DK - Denmark | 0.323084453 | 0.035020679 | 9.22553374 | 2.82152E-20 | *** |
| IE - Ireland | -0.154241947 | 0.033953094 | -4.542795089 | 5.55132E-06 | *** |
| GR - Greece | -0.373219087 | 0.035003194 | -10.66242945 | 1.52559E-26 | *** |
| ES -Spain | -0.028692997 | 0.033759362 | -0.849927119 | 0.395365607 |  |
| PT - Portugal | -0.758889546 | 0.036664539 | -20.69818894 | 3.59633E-95 | *** |
| DE-E Germany East | 0.153532466 | 0.047792506 | 3.212479932 | 0.001315943 | ** |
| FI - Finland | 0.568217848 | 0.034317634 | 16.55760581 | 1.41098E-61 | *** |
| SE - Sweden | 0.311490467 | 0.033322569 | 9.34773271 | 8.95466E-21 | *** |
| AT - Austria | -0.051393822 | 0.033807319 | -1.520198117 | 0.12846119 |  |
| CY - Cyprus (Republic) | -0.449458973 | 0.049189929 | -9.137215239 | 6.40811E-20 | *** |
| CZ - Czech Republic | 0.147286186 | 0.032059842 | 4.594102114 | 4.34617E-06 | *** |
| EE - Estonia | 0.409474116 | 0.033362164 | 12.27360796 | 1.25532E-34 | *** |
| HU - Hungary | -0.157212293 | 0.03351676 | -4.690557664 | 2.72461E-06 | *** |
| LV - Latvia | 0.420115968 | 0.033852102 | 12.41033623 | 2.29686E-35 | *** |
| LT - Lithuania | 0.271201772 | 0.034273223 | 7.912934511 | 2.51392E-15 | *** |
| MT - Malta | -0.586000314 | 0.050052948 | -11.70760836 | 1.1652E-31 | *** |
| PL - Poland | -0.610347495 | 0.034366451 | -17.75998061 | 1.44284E-70 | *** |
| SK - Slovakia | 0.269949904 | 0.033867234 | 7.970828177 | 1.57615E-15 | *** |
| SI - Slovenia | 0.214338662 | 0.033156827 | 6.464390037 | 1.01708E-10 | *** |
| BG - Bulgaria | -0.149730938 | 0.033727374 | -4.439448436 | 9.01897E-06 | *** |
| RO - Romania | -0.323956411 | 0.033681246 | -9.618302558 | 6.69264E-22 | *** |
| HR - Croatia | -0.067202239 | 0.033411824 | -2.011331069 | 0.044290499 | * |
| Age15-24 | 0.346972781 | 0.033441771 | 10.37543093 | 3.20762E-25 | *** |
| Age25-34 | 0.121824717 | 0.018539509 | 6.571086452 | 4.99494E-11 | *** |
| Age35-44 | 0.027991038 | 0.015795147 | 1.772129059 | 0.076373136 | . |
| Age45-54 | -0.017762694 | 0.015026861 | -1.182062862 | 0.237180759 |  |
| Age55-64 | -0.051073496 | 0.014536384 | -3.513493797 | 0.000442255 | *** |
| Age65+ | -0.137255186 | 0.012849202 | -10.68200064 | 1.23579E-26 | *** |
| AgeRefusal | -0.384772986 | 0.315394683 | -1.219972962 | 0.222475125 |  |
| LS Very satisfied | 0.123545281 | 0.013249142 | 9.324775861 | 1.11218E-20 | *** |
| LS Fairly satisfied | -0.011659194 | 0.005673056 | -2.055187664 | 0.039860882 | * |
| LS Not very satisfied | -0.139536607 | 0.018130031 | -7.696435312 | 1.39915E-14 | *** |
| LS Not at all satisfied | -0.122352071 | 0.042226182 | -2.897540435 | 0.003761013 | ** |
| LS DK (SPONT.) | -0.087319509 | 0.189340414 | -0.461177342 | 0.644671376 |  |
| EU MB A good thing | 0.010794583 | 0.006039632 | 1.787291421 | 0.0738904 | . |
| EU MB A bad thing | -0.006147137 | 0.025302353 | -0.242947236 | 0.808046273 |  |
| EU MB Neither a good thing nor a bad thing | -0.022728891 | 0.012411045 | -1.831343909 | 0.067049228 | . |
| EU MB DK (SPONT.) | -0.071351987 | 0.080616307 | -0.885081317 | 0.376112775 |  |
| EU Future Very optimistic | 0.011194718 | 0.022924288 | 0.488334387 | 0.625313008 |  |
| EU Future Fairly optimistic | 0.018054082 | 0.006739624 | 2.67879683 | 0.007388721 | ** |
| EU Future Fairly pessimistic | -0.015638182 | 0.012083435 | -1.294183492 | 0.195602048 |  |
| EU Future Very pessimistic | -0.024858575 | 0.029713375 | -0.836612311 | 0.402810523 |  |
| EU Future DK (SPONT.) | -0.147561798 | 0.036511442 | -4.04152208 | 5.31054E-05 | *** |
| Help Refugees Totally agree | 0.057969605 | 0.007646393 | 7.581300491 | 3.42108E-14 | *** |
| Help Refugees Tend to agree | -0.055207336 | 0.008006824 | -6.895035433 | 5.38514E-12 | *** |
| Help Refugees Tend to disagree | -0.042523927 | 0.025397414 | -1.674340814 | 0.094063638 | . |
| Help Refugees Totally disagree | 0.000933698 | 0.039894446 | 0.023404203 | 0.981327853 |  |
| Help Refugees DK (SPONT.) | -0.053588558 | 0.045727973 | -1.171898834 | 0.241237675 |  |
| LR Placement 1 Left | -0.009453014 | 0.031207316 | -0.302910199 | 0.761958299 |  |
| LR Placement 2 | 0.043921399 | 0.033450939 | 1.31300947 | 0.189179757 |  |
| LR Placement 3 | 0.024654523 | 0.021190719 | 1.163458547 | 0.244643507 |  |
| LR Placement 4 | -0.000373057 | 0.020402686 | -0.018284697 | 0.985411735 |  |
| LR Placement 5 | 0.003092433 | 0.011298213 | 0.2737099 | 0.784307569 |  |
| LR Placement 6 | -0.023610542 | 0.018677421 | -1.264122193 | 0.206186172 |  |
| LR Placement 7 | 0.011230353 | 0.019369623 | 0.579792011 | 0.562054885 |  |
| LR Placement 8 | -0.028505227 | 0.023348017 | -1.220884273 | 0.22212984 |  |
| LR Placement 9 | 0.023106189 | 0.040875137 | 0.565287133 | 0.571878479 |  |
| LR Placement 10 Right | -0.034006028 | 0.032213194 | -1.055655271 | 0.29112574 |  |
| LR Placement Refusal (SPONT.) | 0.03675775 | 0.030216975 | 1.216460288 | 0.22380963 |  |
| LR Placement DK (SPONT.) | -0.023397677 | 0.025933107 | -0.902231901 | 0.366933692 |  |
| Marital Status (Re)Married | 0.009165842 | 0.007086797 | 1.2933689 | 0.195883501 |  |
| Marital Status Single living with a partner | 0.028645551 | 0.020666492 | 1.386086771 | 0.165720409 |  |
| Marital Status Single | 0.039612647 | 0.015911437 | 2.489570756 | 0.012789746 | * |
| Marital Status Divorced or separated | 0.032161676 | 0.022793458 | 1.411004684 | 0.158243233 |  |
| Marital Status Widow | -0.186148696 | 0.023118713 | -8.051862385 | 8.15436E-16 | *** |
| Marital Status Other (SPONTANEOUS) | -0.105813467 | 0.103023594 | -1.027079938 | 0.304382824 |  |
| Marital Status Refusal (SPONTANEOUS) | 0.075578264 | 0.211436171 | 0.357451914 | 0.720753519 |  |
| Education 15- | -0.214533217 | 0.02169177 | -9.890074332 | 4.59686E-23 | *** |
| Education 16-19 | -0.044562701 | 0.008564419 | -5.203236705 | 1.95847E-07 | *** |
| Education 20+ | 0.105727531 | 0.010073064 | 10.49606474 | 9.00566E-26 | *** |
| Education Still Studying | 0.155452008 | 0.034853963 | 4.460095626 | 8.19231E-06 | *** |
| Education No full-time education | -0.397570329 | 0.081272303 | -4.891830474 | 9.99025E-07 | *** |
| Education Refusal | -0.206706937 | 0.092660226 | -2.230805454 | 0.025694019 | * |
| Education Don't know | -0.217589803 | 0.047695795 | -4.562033285 | 5.06606E-06 | *** |
| Man | 0.064174078 | 0.007243546 | 8.859483575 | 8.03859E-19 | *** |
| Woman | -0.054775238 | 0.00628222 | -8.719088875 | 2.80449E-18 | *** |
| Non binary | -0.690370566 | 0.250018282 | -2.761280338 | 0.005757523 | ** |
| Rural area or village | 0.073342547 | 0.012389456 | 5.919755418 | 3.22421E-09 | *** |
| Small/middle town | -0.011978044 | 0.009453492 | -1.26704972 | 0.205137512 |  |
| Large town | -0.065624824 | 0.013874226 | -4.729981079 | 2.24541E-06 | *** |
| Diff. Paying bills Most of the time | -0.087123397 | 0.025257752 | -3.449372565 | 0.000561891 | *** |
| Diff. Paying bills From time to time | -0.030468902 | 0.012195702 | -2.498331066 | 0.01247796 | * |
| Diff. Paying bills Almost never/never | 0.022487789 | 0.005461594 | 4.117440602 | 3.83103E-05 | *** |
| Diff. Paying bills Refusal (SPONT.) | -0.073521261 | 0.077419952 | -0.949642293 | 0.342294041 |  |
| Social class The working class of society | -0.091546665 | 0.013082182 | -6.997812981 | 2.59989E-12 | *** |
| Social class The lower middle class of society | -0.053511966 | 0.016078016 | -3.328269316 | 0.000873873 | *** |
| Social class The middle class of society | 0.040769703 | 0.006916349 | 5.894685241 | 3.75396E-09 | *** |
| Social class The upper middle class of society | 0.121601206 | 0.024224411 | 5.019779667 | 5.17308E-07 | *** |
| Social class The higher class of society | 0.182091744 | 0.070185235 | 2.594445172 | 0.009474372 | ** |
| Social class Other (SPONT.) | -0.286599807 | 0.18964975 | -1.511205825 | 0.130736019 |  |
| Social class None (SPONT.) | 0.074461734 | 0.090210717 | 0.825420039 | 0.409133162 |  |
| Social class Refusal (SPONT.) | 0.10573587 | 0.137458343 | 0.769221188 | 0.441762014 |  |
| Social class DK (SPONT.) | -0.218195469 | 0.070054553 | -3.1146508 | 0.001841628 | ** |
| EU Future. Life NextGen Easier | 0.050701175 | 0.015175496 | 3.340989712 | 0.000834803 | *** |
| EU Future. Life NextGen More difficult | 0.011634005 | 0.006463736 | 1.799888529 | 0.071878241 | . |
| EU Future. Life NextGen About the same | -0.056976501 | 0.01143822 | -4.981238261 | 6.31787E-07 | *** |
| EU Future. Life NextGen DK (SPONT.) | -0.022932779 | 0.044267625 | -0.518048554 | 0.604424393 |  |
| Cities/urban | -0.003629334 | 0.011688697 | -0.310499421 | 0.7561812 |  |
| Towns/suburbs | 0.006569769 | 0.010392509 | 0.632163833 | 0.527279828 |  |
| Rural | -0.002563516 | 0.013830709 | -0.18534958 | 0.852954853 |  |
| Threshold (1->2) | -0.95358838 | 0.009395336 | -101.4959357 | 0 | *** |
| Threshold (2->3) | -0.278193281 | 0.008159128 | -34.09595575 | 8.4678E-255 | *** |
| Threshold (3->4) | 0.302623478 | 0.008149893 | 37.13220249 | 8.4949E-302 | *** |
| Threshold (4->5) | 0.952869112 | 0.009272283 | 102.7653178 | 0 | *** |
| Discussion: *** (p<0.001); ** (p<0.01); * (p<0.05); . (p<0.1) | | | | | |

Table A2. Marginal effects for being in the first quintile of PASI (Sedentaries)

| **Variable** | **Marg..Eff** | **Std..error** | **t.value** | **Pr...t..** | **Disc** |
| --- | --- | --- | --- | --- | --- |
| FR - France | 0.010793183 | 0.008397294 | 1.285316716 | 0.198681631 |  |
| BE - Belgium | 0.024635165 | 0.00812401 | 3.032389655 | 0.002426258 | ** |
| NL - The Netherlands | -0.109162858 | 0.008814141 | -12.38496782 | 3.15211E-35 | *** |
| DE-W - Germany - West | -0.057120656 | 0.008464941 | -6.747909598 | 1.4999E-11 | *** |
| IT - Italy | 0.102115986 | 0.008707894 | 11.72682916 | 9.28729E-32 | *** |
| LU - Luxembourg | -0.033869816 | 0.012127889 | -2.792721363 | 0.005226669 | ** |
| DK - Denmark | -0.081802665 | 0.008880736 | -9.211247882 | 3.22355E-20 | *** |
| IE - Ireland | 0.039052954 | 0.008599336 | 4.541391901 | 5.5884E-06 | *** |
| GR - Greece | 0.094496395 | 0.008881118 | 10.64014582 | 1.93826E-26 | *** |
| ES -Spain | 0.007264861 | 0.008547298 | 0.849959963 | 0.395347346 |  |
| PT - Portugal | 0.192145387 | 0.009376475 | 20.49228347 | 2.52279E-93 | *** |
| DE-E Germany East | -0.038873319 | 0.01210347 | -3.211749929 | 0.001319292 | ** |
| FI - Finland | -0.143868681 | 0.008731771 | -16.47646101 | 5.41641E-61 | *** |
| SE - Sweden | -0.078867151 | 0.008449425 | -9.334026286 | 1.01924E-20 | *** |
| AT - Austria | 0.013012547 | 0.008559936 | 1.520168644 | 0.128468596 |  |
| CY - Cyprus (Republic) | 0.113799786 | 0.012476063 | 9.121449845 | 7.41259E-20 | *** |
| CZ - Czech Republic | -0.037291805 | 0.008120331 | -4.592399434 | 4.38179E-06 | *** |
| EE - Estonia | -0.103675907 | 0.008472836 | -12.23627012 | 1.98968E-34 | *** |
| HU - Hungary | 0.039805024 | 0.008489795 | 4.688572976 | 2.75117E-06 | *** |
| LV - Latvia | -0.106370348 | 0.008596827 | -12.37321053 | 3.64937E-35 | *** |
| LT - Lithuania | -0.068666342 | 0.008689158 | -7.902530801 | 2.73297E-15 | *** |
| MT - Malta | 0.148371074 | 0.01270294 | 11.68005774 | 1.61186E-31 | *** |
| PL - Poland | 0.154535606 | 0.008759382 | 17.64229628 | 1.16624E-69 | *** |
| SK - Slovakia | -0.068349378 | 0.008585597 | -7.960934892 | 1.70744E-15 | *** |
| SI - Slovenia | -0.054269011 | 0.00840203 | -6.459035508 | 1.05372E-10 | *** |
| BG - Bulgaria | 0.037910799 | 0.008541781 | 4.438278108 | 9.06814E-06 | *** |
| RO - Romania | 0.082023438 | 0.008541331 | 9.603121952 | 7.75589E-22 | *** |
| HR - Croatia | 0.017015125 | 0.008459992 | 2.011245958 | 0.044299484 | * |
| Age15-24 | -0.087851018 | 0.008484242 | -10.35461035 | 3.98807E-25 | *** |
| Age25-34 | -0.030845144 | 0.004697625 | -6.566115238 | 5.16449E-11 | *** |
| Age35-44 | -0.00708713 | 0.003999436 | -1.77203252 | 0.076389159 | . |
| Age45-54 | 0.004497387 | 0.003804796 | 1.182030818 | 0.237193473 |  |
| Age55-64 | 0.012931443 | 0.003681371 | 3.512670626 | 0.000443627 | *** |
| Age65+ | 0.034752028 | 0.003259979 | 10.66019995 | 1.56261E-26 | *** |
| AgeRefusal | 0.097421759 | 0.079858323 | 1.219932433 | 0.22249049 |  |
| LS Very satisfied | -0.031280779 | 0.003360251 | -9.309058995 | 1.2897E-20 | *** |
| LS Fairly satisfied | 0.002952024 | 0.001436449 | 2.055084457 | 0.039870848 | * |
| LS Not very satisfied | 0.035329668 | 0.004596286 | 7.686567868 | 1.51135E-14 | *** |
| LS Not at all satisfied | 0.030978666 | 0.010693359 | 2.89700055 | 0.003767491 | ** |
| LS DK (SPONT.) | 0.022108673 | 0.047939806 | 0.461175687 | 0.644672564 |  |
| EU MB A good thing | -0.002733111 | 0.00152928 | -1.787188303 | 0.073907059 | . |
| EU MB A bad thing | 0.001556411 | 0.006406367 | 0.2429475 | 0.808046068 |  |
| EU MB Neither a good thing nor a bad thing | 0.005754792 | 0.003142599 | 1.831220636 | 0.067067618 | . |
| EU MB DK (SPONT.) | 0.018065811 | 0.020411963 | 0.885059974 | 0.376124286 |  |
| EU Future Very optimistic | -0.002834422 | 0.005804332 | -0.488328797 | 0.625316967 |  |
| EU Future Fairly optimistic | -0.004571164 | 0.001706729 | -2.678317903 | 0.007399295 | ** |
| EU Future Fairly pessimistic | 0.003959476 | 0.003059603 | 1.294114165 | 0.195625989 |  |
| EU Future Very pessimistic | 0.006294013 | 0.007523311 | 0.8366015 | 0.402816602 |  |
| EU Future DK (SPONT.) | 0.037361588 | 0.009248256 | 4.039852423 | 5.34848E-05 | *** |
| Help Refugees Totally agree | -0.014677488 | 0.001938308 | -7.572321146 | 3.66614E-14 | *** |
| Help Refugees Tend to agree | 0.013978101 | 0.002029362 | 6.887929178 | 5.66104E-12 | *** |
| Help Refugees Tend to disagree | 0.010766753 | 0.006430762 | 1.674257832 | 0.094079938 | . |
| Help Refugees Totally disagree | -0.000236406 | 0.01010099 | -0.023404197 | 0.981327858 |  |
| Help Refugees DK (SPONT.) | 0.013568238 | 0.011578281 | 1.171869867 | 0.241249307 |  |
| LR Placement 1 Left | 0.002393435 | 0.007901466 | 0.302910302 | 0.76195822 |  |
| LR Placement 2 | -0.011120583 | 0.008469721 | -1.312981008 | 0.189189348 |  |
| LR Placement 3 | -0.006242348 | 0.00536539 | -1.163447309 | 0.244648064 |  |
| LR Placement 4 | 9.44553E-05 | 0.005165814 | 0.018284695 | 0.985411737 |  |
| LR Placement 5 | -0.000782982 | 0.002860641 | -0.273708482 | 0.784308659 |  |
| LR Placement 6 | 0.00597802 | 0.004729112 | 1.264089406 | 0.206197939 |  |
| LR Placement 7 | -0.002843445 | 0.004904233 | -0.579793935 | 0.562053588 |  |
| LR Placement 8 | 0.007217319 | 0.005911741 | 1.220844916 | 0.222144744 |  |
| LR Placement 9 | -0.005850321 | 0.01034949 | -0.565276266 | 0.571885869 |  |
| LR Placement 10 Right | 0.008610082 | 0.008156344 | 1.055630074 | 0.291137255 |  |
| LR Placement Refusal (SPONT.) | -0.009306799 | 0.007651204 | -1.216383457 | 0.223838883 |  |
| LR Placement DK (SPONT.) | 0.005924124 | 0.006566289 | 0.902202703 | 0.366949199 |  |
| Marital Status (Re)Married | -0.002320726 | 0.001794386 | -1.293325804 | 0.1958984 |  |
| Marital Status Single living with a partner | -0.007252848 | 0.005232736 | -1.386052822 | 0.165730775 |  |
| Marital Status Single | -0.010029638 | 0.004029324 | -2.489161306 | 0.012804486 | * |
| Marital Status Divorced or separated | -0.008143106 | 0.005771505 | -1.410915381 | 0.158269566 |  |
| Marital Status Widow | 0.047131514 | 0.005862344 | 8.039704862 | 9.00551E-16 | *** |
| Marital Status Other (SPONTANEOUS) | 0.02679121 | 0.026085449 | 1.027055751 | 0.304394213 |  |
| Marital Status Refusal (SPONTANEOUS) | -0.019135874 | 0.053534234 | -0.357451159 | 0.720754084 |  |
| Education 15- | 0.054318271 | 0.005504467 | 9.868035518 | 5.72753E-23 | *** |
| Education 16-19 | 0.011282956 | 0.002168917 | 5.202115834 | 1.97032E-07 | *** |
| Education 20+ | -0.026769452 | 0.002555502 | -10.47522054 | 1.12276E-25 | *** |
| Education Still Studying | -0.039359333 | 0.008827222 | -4.458858488 | 8.23973E-06 | *** |
| Education No full-time education | 0.100661954 | 0.020588662 | 4.889193494 | 1.0125E-06 | *** |
| Education Refusal | 0.052336713 | 0.023462962 | 2.230609829 | 0.025706985 | * |
| Education Don't know | 0.055092176 | 0.01208168 | 4.559976429 | 5.11594E-06 | *** |
| Man | -0.016248416 | 0.001836818 | -8.845960177 | 9.07446E-19 | *** |
| Woman | 0.013868697 | 0.00159297 | 8.706190562 | 3.14258E-18 | *** |
| Non binary | 0.174796873 | 0.063311932 | 2.760883565 | 0.005764522 | ** |
| Rural area or village | -0.018569806 | 0.003138934 | -5.915959205 | 3.29947E-09 | *** |
| Small/middle town | 0.003032755 | 0.002393668 | 1.266990322 | 0.20515875 |  |
| Large town | 0.016615735 | 0.003514108 | 4.728294007 | 2.26414E-06 | *** |
| Diff. Paying bills Most of the time | 0.022059019 | 0.006397198 | 3.448231329 | 0.000564271 | *** |
| Diff. Paying bills From time to time | 0.007714507 | 0.003088063 | 2.498170497 | 0.012483614 | * |
| Diff. Paying bills Almost never/never | -0.005693747 | 0.00138327 | -4.116151334 | 3.85251E-05 | *** |
| Diff. Paying bills Refusal (SPONT.) | 0.018615056 | 0.01960259 | 0.949622247 | 0.34230423 |  |
| Social class The working class of society | 0.023178959 | 0.003314768 | 6.992633372 | 2.69774E-12 | *** |
| Social class The lower middle class of society | 0.013548846 | 0.004071837 | 3.327452616 | 0.000876439 | *** |
| Social class The middle class of society | -0.010322596 | 0.001752264 | -5.8910067 | 3.8385E-09 | *** |
| Social class The upper middle class of society | -0.030788553 | 0.006135954 | -5.017728699 | 5.22859E-07 | *** |
| Social class The higher class of society | -0.046104323 | 0.017772512 | -2.594136571 | 0.009482881 | ** |
| Social class Other (SPONT.) | 0.072565014 | 0.048020871 | 1.511114095 | 0.130759384 |  |
| Social class None (SPONT.) | -0.018853177 | 0.02284096 | -0.825410863 | 0.40913837 |  |
| Social class Refusal (SPONT.) | -0.026771564 | 0.034803613 | -0.769217942 | 0.441763941 |  |
| Social class DK (SPONT.) | 0.055245527 | 0.017740913 | 3.114018178 | 0.001845581 | ** |
| EU Future. Life NextGen Easier | -0.012837174 | 0.003843374 | -3.340079154 | 0.000837545 | *** |
| EU Future. Life NextGen More difficult | -0.002945647 | 0.001636683 | -1.799765627 | 0.071897654 | . |
| EU Future. Life NextGen About the same | 0.014426041 | 0.002897782 | 4.978304743 | 6.41436E-07 | *** |
| EU Future. Life NextGen DK (SPONT.) | 0.005806415 | 0.011208252 | 0.51804824 | 0.604424613 |  |
| Cities/urban | 0.000918921 | 0.002959504 | 0.310498409 | 0.756181969 |  |
| Towns/suburbs | -0.001663418 | 0.002631349 | -0.632154153 | 0.527286152 |  |
| Rural | 0.000649064 | 0.003501842 | 0.18534928 | 0.852955088 |  |
| Discussion: *** (p<0.001); ** (p<0.01); * (p<0.05); . (p<0.1) | | | | | |
